# The Impact of Vertical Sleeve Gastrectomy on Behavioral and Reward‐Related Outcomes

**DOI:** 10.1002/brb3.71766

**Published:** 2026-09-14

**Authors:** Jayasinghe R, Piotrowski J, Greaves E, Lemus MB, Reed F, Conn K, Brown RM, Sumithran P, Andrews ZB, Foldi CJ, Oldfield BJ, Stefanidis A

**Affiliations:** ^1^ Department of Physiology Monash University Clayton VIC Australia; ^2^ Biomedicine Discovery Institute Monash University Clayton VIC Australia; ^3^ Australian Eating Disorders Research & Translation Centre (AEDRTC) Sydney NSW Australia; ^4^ Florey Institute of Neuroscience and Mental Health Parkville VIC Australia; ^5^ Department of Biochemistry and Pharmacology Melbourne University Parkville VIC Australia; ^6^ Department of Surgery, School of Translational Medicine Monash University Melbourne VIC Australia; ^7^ Department of Endocrinology and Diabetes Alfred Health Melbourne VIC Australia; ^8^ Institute For Health Transformation, Faculty of Health Deakin University Geelong Victoria Australia

## Abstract

**Introduction:**

Vertical sleeve gastrectomy (VSG) is widely recognized as an effective metabolic surgery for achieving durable weight loss and improving obesity‐related comorbidities. However, some patients experience deteriorating mental health post‐surgery, prompting investigation into underlying mechanisms.

**Methods:**

This study examined diet‐induced obese C57BL/6J male mice that underwent either VSG or sham surgery, as well as a cohort of high‐fat diet‐fed male mice subsequently transitioned to a low‐fat diet (LFD), compared to chow‐fed controls. We assessed metabolic parameters (body weight, food intake, glucose tolerance, and body composition), interscapular temperature, a proxy measure of thermogenic activity via transponder‐monitored activity and temperature in brown and white adipose tissue and behavioral outcomes related to hedonic processing, anxiety‐like behavior, motivation, and cognitive flexibility.

**Results:**

VSG produced rapid and sustained reductions in body weight, transient decreases in food intake, reduced adiposity, trends toward improved glucose regulation, and increased interscapular adipose tissue temperature, suggesting increased brown adipose tissue thermogenic activity. Preferences for sucrose and saccharin remained unchanged in the low‐fat diet‐fed cohort. VSG reduced responding to rewards under high‐fat diet conditions, an effect not observed in a separate cohort of mice transitioned to a LFD post‐surgery but did not significantly enhance cognitive flexibility. In contrast, sham‐operated low‐fat diet‐fed mice demonstrated improved cognitive flexibility. Anxiety‐like behaviors were unaffected by VSG.

**Conclusions:**

These findings confirm the robust metabolic benefits of VSG. Exploratory comparisons between independent dietary cohorts suggest that dietary context may influence reward‐related and cognitive outcomes following VSG. However, these findings are based on comparisons between separate dietary cohorts and should be interpreted cautiously. Further studies are required to determine whether dietary context contributes to long‐term behavioral outcomes following VSG.

## Graphical abstract

Vertical sleeve gastrectomy (VSG) produces robust and consistent metabolic improvements in diet‐induced obese male mice. However, post‐surgical behavioral, reward‐related, and cognitive outcomes may be dependent on dietary context, as VSG reduces operant responding for sucrose under high‐fat diet maintenance but yields no significant behavioral changes under low‐fat diet conditions.

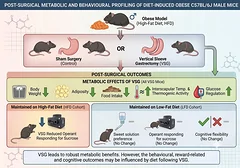

## 1 Introduction

The rising prevalence of obesity in children and adults is a major global health concern (Chang et al. 2014; Afshin et al. 2017; Smith and Smith 2016), affecting up to 35% of adults in some developed countries (Afshin et al. 2017; Smith and Smith 2016). Obesity is associated with numerous comorbidities including type 2 diabetes mellitus (T2DM), hypertension, dyslipidaemia, cardiovascular disease, some forms of cancer and mental health issues such as anxiety and depression (Pi‐Sunyer 1999; Abdelaal et al. 2017; Frühbeck et al. 2013).

Metabolic bariatric surgery has long been recognized as the most efficacious treatment to achieve sustained weight loss, often by 20%–30% (Stefanidis and Oldfield 2017; Lee and Dixon 2017), reductions in adiposity, and improvements in obesity‐related comorbidities. Vertical sleeve gastrectomy (VSG) is currently the most commonly performed procedure (Lewis et al. 2021; (ANZBSR) AaNZBSR 2025). By removing approximately 80% of the stomach, VSG reduces gastric capacity, alters gut hormone release, activates vagal sensory circuits, changes gut microbiota, all of which may contribute to rapid and sustained satiety (Evers et al. 2017).

In addition to improvements in metabolic health, there are reports that VSG leads to improvements in psychological health; however, there is a subset of patients who report a deterioration in mental health status following surgery (Castaneda et al. 2019). In most cases, mental health issues have been reported to emerge 2–3 years post‐surgery (Sjöström et al. 2004), with depression rates rebounding or exceeding pre‐surgical levels, an increase in the use of psychotropic medication, increased risk of alcohol and substance use disorders and an alarming 4–5 fold increase in the risk of suicide or self‐harm (Castaneda et al. 2019; Tindle et al. 2010; Guerrero‐Hreins et al. 2021). VSG has also been found to enhance alcohol and opioid absorption, which likely contributes to developing addictive behaviors (Castaneda et al. 2019). These outcomes have linked to altered gut–brain signaling including reduced ghrelin and increased glucagon like peptide 1 (GLP‐1), cholecystokinin (CCK), and peptide YY (PYY), which have been implicated in the development of depression and anxiety (Castaneda et al. 2019; Manning et al. 2014). Elevated PYY, for example, dampens activation of brain reward pathways, reducing motivation for food (Castaneda et al. 2019; Roerig et al. 2013; Scholtz et al. 2014). VSG has also been shown to alter dopamine release in the ventral striatum (VS), a key site for reward processing and motivation (Halbout et al. 2019; Liljeholm and O'Doherty 2012). While clinical reports of suicide, self‐harm, and substance‐use disorders provide important context, the behavioral assays used in this study assess preclinical constructs related to reward processing, motivation, cognitive flexibility, and anxiety‐like behavior, and should not be interpreted as direct models of these outcomes.

This study aimed to characterize the effect of VSG on metabolic, reward‐related and anxiety‐like outcomes in a mouse model. We hypothesized that VSG would improve metabolic parameters while reducing motivation for palatable foods and altering anxiety‐like behavior and cognitive function.

## 2 Materials and Methods

### 2.1 Animals

All experiments were conducted in adult male C57BL/6J mice (*n* = 78) that were individually housed in a temperature (25°C ± 1°C) and humidity‐controlled environment under a standard 12‐h:12‐h light/dark cycle with the lights turning on at 0700 h and off at 1900 h and had ad libitum access to food throughout the experimental periods unless specified otherwise. Mice that were due to receive either VSG or sham surgery were subjected to diet‐induced obesity (DIO) by providing them with a high‐fat diet (HFD; 43% calories from lipids, Specialty Feeds, SFO4‐001, WA Australia) for 14–20 weeks, starting from the age of 4 weeks. The mice that were eventually placed in the control group received a standard chow diet (either 8.5% kcal from lipids, Barastoc Ridley, Victoria, Australia or 12% kcal from lipids, Specialty Feeds, Irradiated rat and mouse diet, WA Australia) for the same period. Body weights and food intake were measured regularly throughout the study period. All experiments in our study were conducted in accordance with the *Australian Code of Practice for the Care and Use of Animals for Scientific Purposes* and approved by the Monash Animal Research Platform Ethics Committee, Clayton, Victoria, Australia (MARP‐1 AEC; 17793, 37482, and 37794).

### 2.2 Experimental Design, Randomization, and Blinding

IO mice were randomly assigned to one of two experimental groups: VSG or sham surgery. Investigators performing behavioral testing, metabolic measurements, and data analysis were blinded to group allocation wherever possible. Surgeries were conducted by a separate investigator who was not involved in subsequent behavioral testing or data analysis. Behavioral testing was quantified using an automated tracking software (EthoVision XT, Noldus Information Technology), minimizing observer bias during behavioral scoring.

Chow‐fed control (CF‐control) mice were included as a physiological reference for baseline metabolic and behavioral responses in non‐obese animals. These mice were not subjected to surgery, and in the HFD experiments, body weight and food intake of CF‐controls were not measured. In the LFD experiments, CF‐control body weight and food intake were recorded only after the transition to the low‐fat diet (LFD). They were included in statistical comparisons to demonstrate normal metabolic and behavioral responses in the absence of DIO or surgical intervention. Male mice were used exclusively to facilitate direct comparison with prior research conducted in male animals.

Sample sizes were based on previous VSG studies conducted in our laboratory. No formal a priori power analysis was conducted in this study. The HFD‐maintained cohort was intended as an exploratory pilot cohort to assess metabolic and behavioral outcomes following VSG, whereas the LFD cohorts were subsequently used to investigate these outcomes under conditions that more closely resemble clinical dietary management following VSG.

#### 2.2.1 Validating and Characterizing the Metabolic and Behavioral Outcomes of VSG

In the validation study, DIO mice were randomly assigned to either the VSG‐ or sham surgery group (*n* = 7 and *n* = 6, respectively) and were maintained on the HFD throughout the entire experimental period, while the CF‐control group (*n* = 5) was maintained on standard chow (8.5% kcal from lipids, Barastoc Ridley, Victoria, Australia. One CF‐control mouse was excluded from energy expenditure analyses due to failure of the implanted telemetry transponder to record temperature data. This mouse was subsequently euthanized for welfare reasons and was therefore also excluded from behavioral analyses, resulting in a final control group size of *n*  = 4 for energy expenditure and behavioral outcomes.

#### 2.2.2 Characterizing the Metabolic and Behavioral Outcomes of VSG Following the Transition to a Low‐Fat Diet

Two independent cohorts of mice (total *n* = 60) were used to investigate the metabolic and behavioral impacts of VSG whilst consuming a LFD. Cohorts were conducted sequentially under identical experimental conditions, including housing environment, light–dark cycle, animal handling, and behavioral testing protocols. Approximately 3–4 weeks following surgery, VSG‐operated (*n* = 23), sham‐operated (*n* = 17), and CF‐control mice (*n* = 20) were gradually transitioned to a LFD (12.3% kcal from lipids, Specialty Feeds, SF13‐081, WA Australia) that was nutritionally matched to the HFD used previously, following a 3‐day food preference period where the new diet was introduced in the presence of their original diets. Experiments commenced 2–3 weeks after this transition to ensure acclimatization to the diet and stabilization of food intakes and body weights. Data from the two cohorts were initially analyzed separately and showed similar patterns of metabolic and behavioral outcomes. Therefore, the cohorts were pooled for the purpose of analyzing metabolic and behavioral outcomes that were conducted under identical conditions. The cohorts were then differentiated at the level of downstream operant tasks to avoid cross‐task interference. The first cohort was assigned to undergo a progressive ratio task (PRT) and the second was subjected to a reversal learning task (RLT).

### 2.3 Surgical Procedures

#### 2.3.1 VSG and Sham Surgery

Following an overnight fast starting toward the end of the light phase, mice were anaesthetized using 5% isoflurane (Isoflurane Inhalation Anaesthetic, Pharmachem, Eagle Farm, Queensland, Australia) in oxygen, and the isoflurane concentration was maintained at 2% throughout surgeries. Mice were administered the anti‐inflammatory drug meloxicam (2 mg/kg subcutaneous, Metacam Solution for Injection, Boehringer Ingelheim, Ingelheim, Germany) and the antibiotics amoxicillin (10 mg/kg subcutaneous, Aspen Phamacare, New South Wales, Australia) and oxytetracycline (Oxytet, 10 mg/kg intraperitoneal, Troy Laboratories Pty. Ltd., New South Wales, Australia) for minimizing pain, inflammation, and the risk of infection. A midline skin incision was made, and a separate incision through the muscle wall allowed access to the stomach, which was externalized and kept moist with sterile saline. The muscle wall incision was closed using a continuous stitch with 6‐0 USP silk braided suture (Look Silk Suture, Surgical Specialties Corporation, PA, USA), and individual suture ties with 5‐0 USP silk braided suture (Look Silk Suture, Surgical Specialties Corporation, PA, USA) were used to close the skin incision. The VSG procedure entailed the removal of approximately 80% of the stomach along the greater curvature to excise the forestomach and the majority of the glandular stomach. While the mice were under steady anesthesia, a LigaClip (LigaClip Extra, Ethicon Endo‐Surgery, USA) was clamped (Ethicon Endo‐Surgery Clamp, J&J Medical Devices, NJ, USA) down along the angle of His, prior to removing the tissue lateral to the LigaClip. The cut edge was further secured using tissue glue (Vetbond Tissue Adhesive, 3 M, NSW, Australia). The sham surgery involved the same abdominal laparotomy to expose the stomach, without the resection of the tissue.

#### 2.3.2 Post‐Operative Care Following VSG and Sham Surgery

Following VSG, sham surgery, or temperature transponder implantation, mice were individually housed and closely monitored throughout a 1‐week recovery period. Immediately after surgery (day 0), mice were fasted overnight. On day 1, the mice received 1 g of HFD pellets placed on the cage floor, followed by 2 g of HFD pellets on day 2. From day 3 onward, mice were returned to ad libitum access to HFD. Body weight, food intake, wound integrity, and general activity were assessed daily. For 5 days post‐surgery, all mice received meloxicam (2 mg/kg, subcutaneous) once daily to minimize pain and inflammation, and amoxicillin (10 mg/kg, subcutaneous) to reduce the risk of infection.

#### 2.3.3 Telemetry Transponder Implantation

Seven days before undergoing VSG and sham surgery, mice underwent implantation of two temperature‐sensing transponders under standard pre‐operative and post‐operative procedures detailed above for VSG and sham surgeries. Anesthesia was induced and maintained using a 2% concentration of isoflurane and meloxicam, amoxicillin and oxytetracycline were administered peri‐operatively. Following aseptic preparation, incisions were made at the left inguinal and interscapular regions. For the interscapular implantation, blunt dissection was used to create a subcutaneous pocket along the midline between the brown adipose tissue (BAT) lobes. A programmable temperature transponder (UID Identification Solutions, IL, USA) was placed such that it was fully encapsulated by the surrounding BAT to enable continuous monitoring of local interscapular temperature, which was used as a proxy of BAT thermogenic activity. For the inguinal implantation, a small incision was made over the left inguinal white adipose tissue (WAT) pad. A pocket was created within the fat depot, and a second temperature transponder was inserted and secured using individual 6‐0 silk suture ties (Look Silk Suture, Surgical Specialties Corporation, PA, USA) to prevent displacement. All skin incisions were closed using standard suturing techniques stated above.

### 2.4 Body Composition Analysis

In the validation study, mice underwent (VSG, *n* = 7; sham‐operated, *n* = 6; CF‐control, *n* = 5) EchoMRI (EchoMRI LLC, Texas, USA) scans that informed us of fat mass, lean mass, and their proportions relative to the total body mass. The EchoMRI system quantifies fat mass, lean mass, total body water, and free water using NMR‐based technology. Each mouse was scanned three times, and the mean of the three scans was used for analysis. Scans were performed 1 day prior to VSG or sham surgery and again at 28 days post‐surgery.

### 2.5 Energy Expenditure Measurements of Brown Adipose Tissue and White Adipose Tissue

Each mouse (VSG, *n* = 7; sham‐operated, *n* = 6 and CF‐control, *n* = 5) was implanted with two transponders (Programmable Microchip with Temperature, UID Identification Solutions, IL, USA) that measure localized temperature and activity; one placed between overlaying BAT lobes in the interscapular region along the midline and the other transponder was positioned within the inguinal white fat pad. This enabled continuous monitoring of interscapular and inguinal adipose tissue temperatures and activity to determine changes in BAT and WAT temperatures. Interscapular temperature was used as a proxy measure of BAT thermogenic activity. Baseline measurements were collected 7 days before surgery. After a 1‐week recovery period, post‐surgical data were recorded continuously for 21 days using the Unified Information Device System on a 48‐h cycle. Average temperature and activity values for each light and dark phase were calculated. Changes in interscapular and inguinal adipose tissue temperature were expressed relative to each animal's baseline.

### 2.6 Glucose and Insulin Tolerance Tests

Glucose tolerance tests (GTT) and an insulin tolerance test (ITT) were conducted 49–56 days after VSG or sham surgery. Mice maintained on HFD (VSG [*n* = 7], sham‐operated [*n* = 6], and CF‐control mice [*n* = 5]) were fasted for 4 h during the light phase and received glucose (2 g/kg per total body weight of 50% wt/vol solution) through oral gavage for the GTT and an intraperitoneal insulin injection (0.75 IU/kg per total body weight) for the ITT. Blood glucose measurements were collected over 120 min using a glucometer (Accu‐Chek Performa II, Roche Diabetes Care) using blood samples collected from a tail vein puncture.

### 2.7 Behavioral Tests

#### 2.7.1 Solution Preference Test

Forty‐one to 43 days after VSG or sham surgery (13–16 days post‐LFD transition), mice maintained on the LFD underwent a 3‐bottle choice test. The first exposure to the solution preference test began approximately 2.5–3 h into the light phase. Whilst having ad libitum access to food, the mice received a choice of 0.5% (w/v) sucrose, 0.02% (w/v) saccharin (Sugarless Liquid Sweetener, Bathox Australia), and water in Falcon conical tubes with sippers. The 0.5% (w/v) sucrose concentration was chosen based on previous studies demonstrating reliable sweet preference while minimizing ceiling effects (Primo et al. 2023). The intake of each solution was measured every 24 h for a period of 72 h. To control for side preference, the positions of the bottles were counterbalanced across cages and treatment groups using six different position combinations (e.g., saccharin left, water middle, and sucrose right), ensuring that each solution appeared in each position across all treatment groups. Total intake for each solution over the 72‐h period was then calculated and expressed as a percentage of the total fluid consumed (water + saccharin + sucrose). Percentage preference for each solution was calculated as: (total volume of a given solution across all six measurements ÷ total volume of all solutions across all six measurements) × 100

#### 2.7.2 Operant Training

Feeding experimentation devices (FED3) were programmed to have a specific active port (either left or right side), and mice were initially allowed to explore the FED and learn that nose poking into the active port delivered a 20 mg sucrose pellet (1811555 [5TUT] Sucrose Pellet, Test Diet, IN, USA) (Matikainen‐Ankney et al. 2021). The training started with 3 days of fixed ratio (FR)‐1 reinforcement schedule, where one active poke resulted in the delivery of a pellet, progressing onto 3 days of FR3 and 3 days of FR5, where 3 or 5 active pokes, respectively, were required to earn a pellet. In total, FR training was conducted over 9–10 days. Mice progressed from FR1 to FR3 to FR5 without strict performance criteria at the lower schedules, as our previous observations indicated that the mice often perform poorly on FR1 and higher ratios are required to improve performance. Once on FR5, mice were maintained on this schedule for 3 days until they reached at least 70% accuracy [(active pokes ÷ total pokes) × 100], after which they advanced to either the PRT or RLT. One sham‐operated mouse in the LFD cohort failed to reach the 70% criterion, as the accuracy reached was approximately 64%. FR training began 2.5 h into the light phase, and each session lasted 2.5 h.

#### 2.7.3 Progressive Ratio Task

Following 9–10 days of FR operant training, mice maintained on the HFD [VSG [*n* = 6], sham‐operated [*n* = 7], and CF‐control mice [*n* = 4]) and a sub cohort of mice maintained on LFD) VSG [*n* = 12], Sham‐operated [*n* = 9], and CF‐control mice [*n* = 10]), underwent 2 separate PRTs on days 73 and 75 post‐VSG and sham surgery (45 and 47 days post‐LFD transition); one under ad libitum feeding and one following food‐restricted conditions. During the PRT, the number of active pokes required to earn subsequent pellets increased progressively, and the exponential schedule increased according to the formula (5 * e(0.2*n)‐ 5), where *n* is the trial number, producing response requirements of 1, 2, 4, 6, 9, 12, etc., to the nearest whole number. The breakpoint was defined as the highest ratio completed. For the fasted condition, food was removed 3.5–4 h before the beginning of the dark phase, resulting in an 18‐h restriction period. PRT testing began 2.5 h into the light phase, and each session was terminated after 2.5 h. The breakpoint was defined as the final ratio completed before session termination. No inactivity timeout or response‐cessation criterion was applied, and therefore no session termination was triggered by behavioral inactivity. The two PRT sessions were separated by 1 day, during which mice were returned to FR5 responding with ad libitum access to food to re‐establish baseline performance before undergoing the food‐restricted PRT the following day.

#### 2.7.4 Reversal Learning Task

A sub‐cohort of mice maintained on LFD (VSG [*n* = 11], sham‐operated [*n* = 8], and CF‐control mice [*n* = 10]) underwent an RLT 64–73 days after VSG and sham surgery (36–45 days post‐LFD transition). Following FR operant training as detailed above, the RLT was designed such that the active port was switched to the opposite side on a FR5 schedule for 3 consecutive days. Following this initial reversal, the active port was switched back to the original configuration. Three serial reversals were carried out in this manner. Each reversal was conducted on the FR5 schedule and spanned across 3 days. The daily session duration was 2.5 h, beginning 2.5 h into the light phase and mice had ad libitum access to LFD throughout testing.

#### 2.7.5 Elevated Plus Maze

Anxiety‐like behavior was assessed in all HFD‐fed and LFD‐fed mice using a standard elevated plus maze (EPM) consisting of two open arms and two closed arms, at approximately 79 days after VSG and sham surgery (51 days post‐LFD transition for the relevant groups). Each arm measured 67.5 cm in length, with closed‐arm walls 16 cm high. The maze was elevated 43 cm above the floor, and the central zone measured 5 × 5 cm. Mice were placed in the center facing an open arm and allowed to explore freely for 10 min. All testing commenced approximately 2.5– 3 h into the light phase, following acclimatization to the behavioral testing room, under dim, non‐aversive lighting conditions (50 lux) routinely used in our facility.

#### 2.7.6 Open Field Test

The open field test (OFT) was used to assess anxiety‐like responses, exploratory behavior, and locomotor behavior. Testing was conducted in a standard rectangular arena (45 × 30 cm, opaque plastic). The center zone was defined as the central 25% of the arena floor. Lighting in the testing room was dimmed to reduce aversive stimulation and kept consistent across all animals. Mice were placed at the center of the apparatus and allowed to explore freely for 10 min. All testing occurred approximately 2.5–3 h into the light phase, following acclimatization to the behavioral testing room, under the same lighting conditions. The OFT was conducted on the day following the EPM, in line with common anxiety‐test battery protocols.

### 2.8 Data Analysis

FED3 operant data were processed using Python (Anaconda Distribution; Anaconda Inc.) using standard FED3 output files to quantify operant performance across fixed ratio, progressive ratio, and reversal learning sessions. Recordings from the EPM and OFT were captured using a Sony HDR‐AS50 camera. Videos were analyzed using EthoVision XT (Noldus Information Technology) for automated tracking and behavioral quantification. Automated tracking reduced experimenter‐related bias by applying consistent detection settings across all animals and sessions. Extracted variables included time spent in predefined zones and total distance travelled.

### 2.9 Statistical Analysis

Data have been presented as mean ± SEM and all statistical analyses were performed using Graphpad Prism 10 (GraphPad Software Inc., San Diego, CA, USA). Comparisons involving two factors were analyzed using two‐way ANOVAs (either repeated measures or mixed‐effects models, as appropriate). Mixed‐effects analyses were applied in cases where the repeated measure data set had missing values, while repeated measures ANOVAs were used when datasets were complete. Missing data were minimal and limited to occasional timepoints in body weight and percentage change in body weight for the HFD‐maintained cohort due to incomplete measurements on specific days. Comparisons involving a single main factor utilized one‐way ANOVAs, and all ANOVAs were followed by post hoc analyses. T‐tests were used where appropriate. *p* < 0.05 was considered to be statistically significant. Detailed statistical analyses, including main effects, interaction effects and post hoc comparisons, are provided in Tables S1–S6.

## 3 Results

### 3.1 Metabolic Benefits of VSG

**FIGURE 1:**
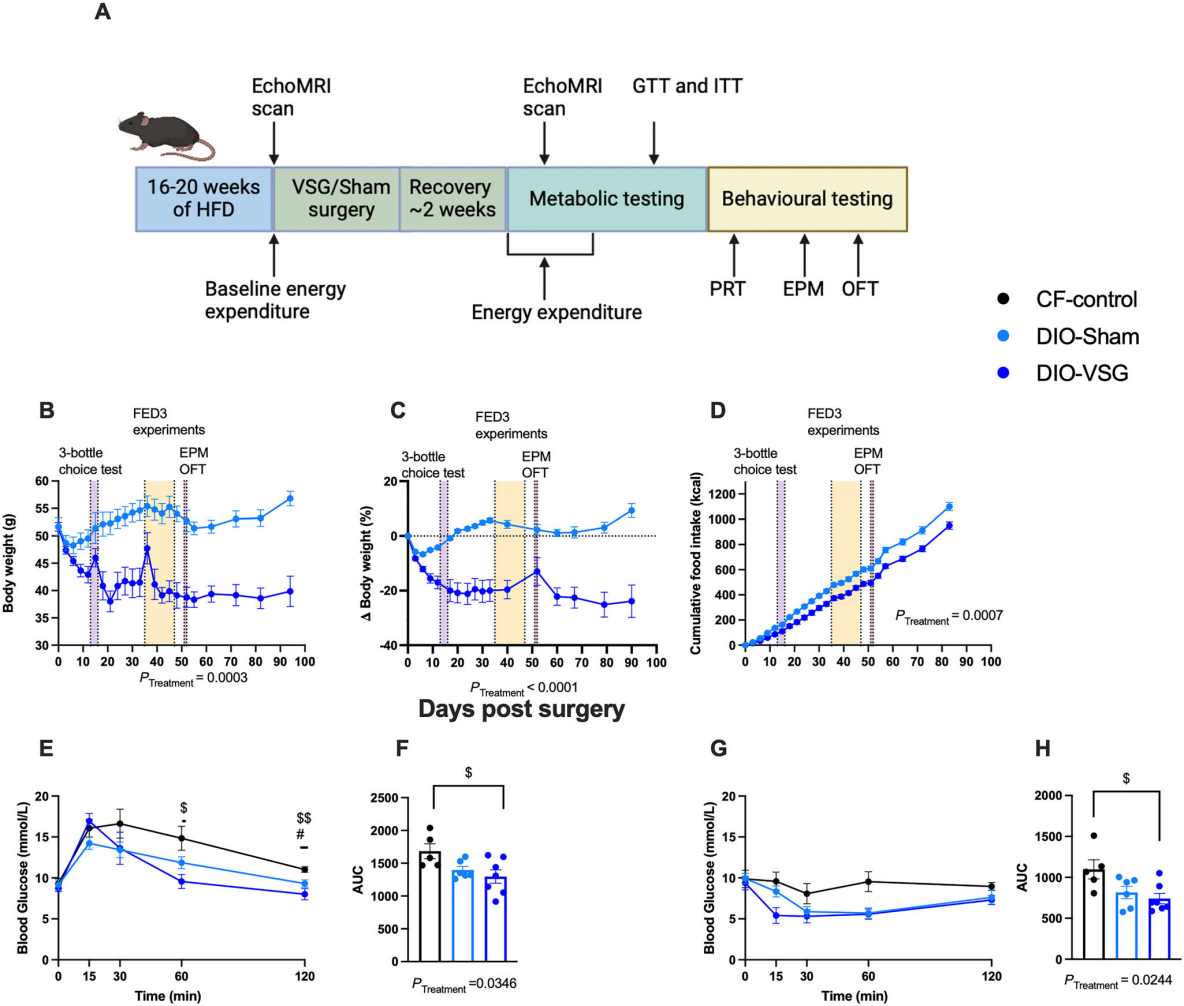
Vertical sleeve gastrectomy (VSG) leads to an overall reduction in body weight, food intake and improved glucose tolerance whilst mice consumed a high‐fat diet. VSG‐ and sham‐operated mice were maintained on a high‐fat diet throughout the experimental period. (A) Experimental protocol, (B,C) Body weights and body weight change (%) over time (*p* < 0.0001), (D) Cumulative food intake (*p* = 0.0007), (E) Blood glucose concentrations following an oral glucose tolerance test (oGTT) (*p* = 0.1076), (F) Area under the curve (AUC) (*p* = 0.0346), (G) Blood glucose concentrations following an insulin tolerance test (ITT) (*p* = 0.0522), (H) Area under the curve (AUC) for the ITT (*p* = 0.0244). Days between dotted lines—days when different experiments and tests indicated in the diagram were conducted. Graphs show mean ± SEM, with bar graphs showing individual animals. Panels B and C were analyzed using two‐way mixed‐effects ANOVAs, panels D, E, and G were analyzed using two‐way repeated measures ANOVAs and panels F and H were analyzed using one‐way ANOVAs. ∗*p* < 0.05, ∗∗*p* < 0.01, ∗∗∗*p* < 0.001, ∗∗∗∗*p* < 0.0001, * = DIO‐VSG versus DIO‐sham‐operated; $ = DIO‐VSG versus chow‐fed control, # = DIO‐sham‐operated versus chow‐fed control. Black: chow‐fed controls (*n* = 5), light blue: DIO‐sham‐operated group (*n* = 6), dark blue: DIO‐VSG‐operated group (*n* = 7). Detailed statistical analyses, including main effects, interaction effects, and post hoc comparisons, are provided in Table S1.

We first evaluated the impact of VSG on body weight and metabolic function in a high‐fat DIO model. VSG resulted in rapid and sustained reductions in body weight. Ten days after VSG, HFD‐fed mice that underwent VSG weighed significantly less than sham‐operated counterparts, with VSG‐operated mice losing 20%–25% of body weight (Figure 1B). As expected, the VSG resulted in a marked reduction in body weight (Figure 1B,C, *P*_Treatment_ < 0.0001), and a consistent decrease in caloric intake, with VSG‐operated mice consuming fewer calories than both sham‐operated mice and CF‐control mice while on the HFD (Figure 1D, *P*_Treatment_ = 0.0007).

To determine the impact of VSG on whole‐body glucose regulation, we performed oGTTs and ITT. Despite the overall effect of the treatment on blood glucose fluctuations over time being non‐significant (Figure 1E, *P*_Treatment_ = 0.1076), mice that underwent VSG surgery displayed a transient increase in blood glucose within the first 15 min, which was followed by a rapid decline. Post hoc comparisons identified differences between treatment groups at 60 min (Figures 1E, *P*_DIO‐VSG vs. CF‐control_ = 0.0430) and 120 min (Figure 1E, *P*_DIO‐VSG vs. CF‐control_ = 0.0098 and *P*_DIO‐sham vs. CF‐control_ = 0.0341). Consistent with these findings, glucose AUC differed between treatment groups (Figure 1F, *P*_Treatment_ = 0.0346). CF‐control mice exhibited slightly reduced glucose tolerance compared to DIO‐sham and DIO‐VSG mice, which may reflect the absence of surgical intervention and limited handling acclimation before testing. VSG showed a non‐significant trend toward improved insulin sensitivity over time in the ITT (Figure 1G, *P*_Treatment_ = 0.0522) and also lower AUC values compared to controls (Figure 1H, *P*_Treatment_ = 0.0244, *P*_DIO‐VSG vs. CF‐control_ = 0.0260).

**FIGURE 2:**
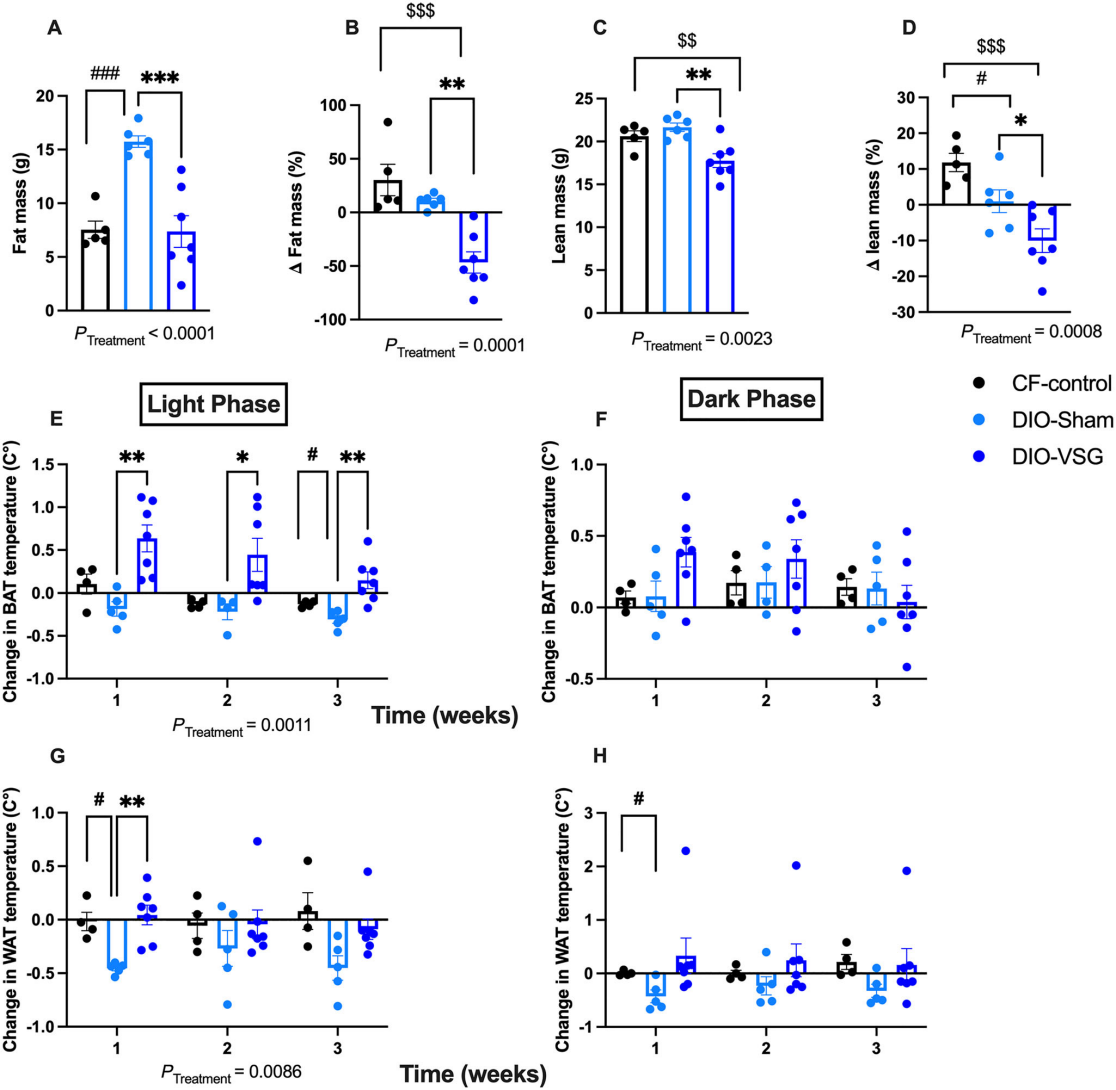
Vertical sleeve gastrectomy (VSG) leads to an overall reduction in body fat mass and improved adipose tissue activity whilst mice consumed a high‐fat diet. VSG‐ and sham‐operated mice were maintained on a high‐fat diet throughout the experimental period. (A) Fat mass (*p* < 0.0001), (B) Change in fat mass (%) (*p* = 0.0001), (C) Lean mass (*p* = 0.0023), (D) Change in lean mass (%) (*p* = 0.0008), (E,F) Change in interscapular temperature, used as a proxy measure of brown adipose tissue (BAT) temperature in the light and dark phases, respectively, (G,H) Change in white adipose tissue (WAT) temperature in the light and dark phases, respectively. Graphs show mean ± SEM, with bar graphs showing individual animals. Panels A–D were analyzed using one‐way ANOVAs and panels E–H were analyzed using two‐way repeated measures ANOVAs. ∗*p* < 0.05, ∗∗*p* < 0.01, ∗∗∗*p* < 0.001, ∗∗∗∗*p* < 0.0001, * = DIO‐VSG versus DIO‐sham‐operated; $ = VSG versus chow‐fed control; # = DIO‐sham‐operated versus chow‐fed control. Black: chow‐fed controls (panels A–D: *n* = 5, panels E–H: *n* = 4), light blue: DIO‐sham‐operated group (*n* = 6), dark blue: DIO‐VSG‐operated group (*n* = 7). Detailed statistical analyses, including main effects, interaction effects, and post hoc comparisons, are provided in Table S2.

Body composition analysis using EchoMRI further corroborated the changes in body weight. VSG resulted in a significant reduction in fat and lean mass (Figure 2A,B, *P*_Treatment_ = 0.0001) relative to baseline (Figure 2C,D, *P*_Treatment_ = 0.0008). To investigate the impact of VSG on energy expenditure, we monitored local temperature changes in interscapular and inguinal adipose tissue depots. VSG significantly increased interscapular temperature during the light phase (Figure 2E, *P*_Treatment_ = 0.0011), suggesting increased BAT thermogenic activity. In contrast, sham‐operated mice tended to exhibit lower WAT temperature compared to CF‐control mice. The evidence for this effect was stronger during the dark phase (Figure 2G, *P*_Treatment_ = 0.0086, *P*_DIO‐sham vs. CF‐control_ = 0.0242). A similar pattern was observed between sham‐operated and CF‐control mice during the light phase, although the overall treatment effect was not statistically significant (Figure 2H, *P*_Treatment_ = 0.2716, *P*_DIO‐sham vs. CF‐control_ = 0.0459), with the difference being most apparent during the first week. This reduction in temperature did not occur following VSG surgery (Figure 2G, *P*_Treatment_ = 0.0086, *P*_DIO‐sham vs. DIO‐VSG_ = 0.0032; Figure 2H, *P*_Treatment_ = 0.2716).

**FIGURE 3:**
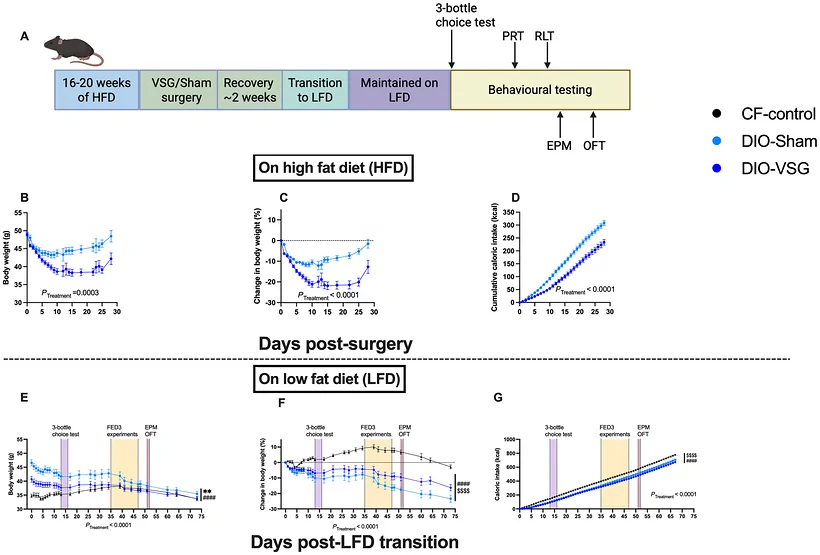
Transitioning to a low‐fat diet can further enhance the weight loss effects of vertical sleeve gastrectomy (VSG). VSG‐ and sham‐operated mice were maintained on a high‐fat diet (HFD) for 28 days and maintained on a low‐fat diet (LFD) for the remainder of the experimental period. (A) Experimental protocol, (B) Body weights whilst on HFD (*p* = 0.0003), (C) Body weight change (%) over time whilst on HFD (*p* < 0.0001), (D) Cumulative HFD intake (*p* = 0.0274), (E) Body weights over time whilst on LFD (*p* < 0.0001), (F) Body weight change (%) over time whilst on LFD (*p* < 0.0001), (G) Cumulative LFD intake (*p* < 0.0001). Days between dotted lines—days when different experiments and tests indicated in the diagram were conducted. Graphs show mean ± SEM and data were analyzed using two‐way repeated measures ANOVAs. VSG versus Sham‐operated; ∗*p* < 0.05, ∗∗*p* < 0.01, ∗∗∗*p* < 0.001, ∗∗∗∗*p* < 0.0001, * = DIO‐VSG versus DIO‐sham‐operated, $ = DIO‐VSG versus chow‐fed control; # = DIO‐sham‐operated versus chow‐fed control. Black: chow‐fed controls (*n* = 20), light blue: DIO‐sham‐operated group (*n* = 17), dark blue: DIO‐VSG‐operated group (*n* = 23). Detailed statistical analyses, including main effects, interaction effects, and post hoc comparisons, are provided in Table S3.

As expected, the weight loss observed following VSG (Figure 3B,C, *P*_Treatment_ = 0.0003 and *P*_Treatment_ < 0.0001, respectively) was further enhanced when mice were transitioned from a HFD to a nutritionally matched LFD, with VSG‐operated animals being lighter than both CF‐control (CF‐control) and sham‐operated groups (Figure 3E,F, *P*_Treatment_ < 0.0001). This reduction in body weight was paralleled by a consistent decrease in caloric intake. VSG‐operated mice consumed fewer calories than both sham‐operated mice and CF‐control mice while on the HFD (Figure 3D, *P*_Treatment_ <0.0001). This effect persisted following the transition to the LFD, where VSG‐operated mice exhibited a lower caloric intake compared to the other two groups, even whilst on the LFD (Figure 3G, *P*_Treatment_ < 0.0001).

### 3.2 The Impact of VSG on Reward‐Related Behavior and Anxiety‐Like Behavior

**FIGURE 4:**
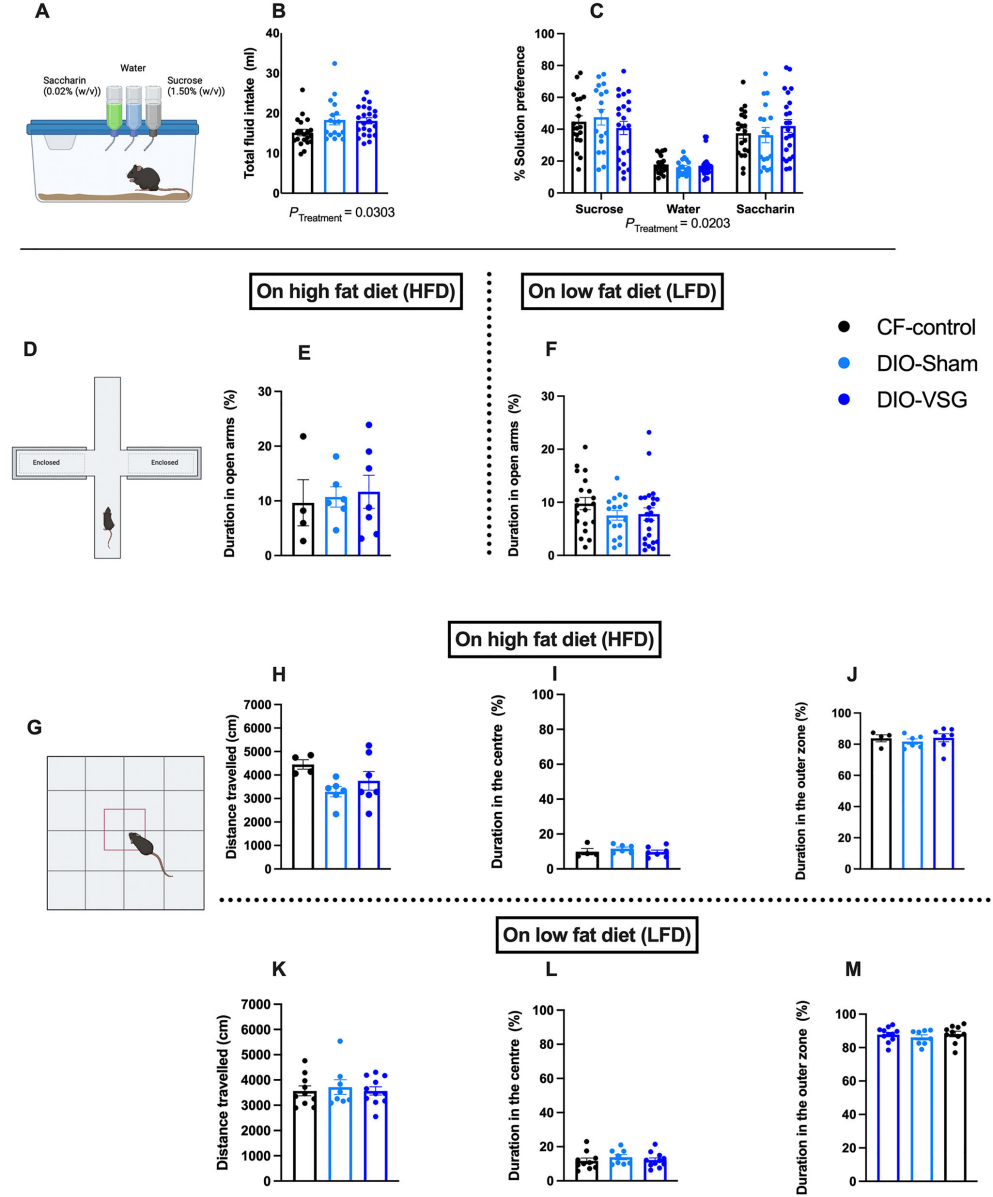
The effect of VSG on reward‐related and anxiety‐like behavior. VSG‐, sham‐operated, and chow‐fed control mice underwent a series of tests to investigate the effects of VSG on various behaviors whilst mice were maintained on LFD—panels B, C, F, K, L, and M and HFD—panels E, H, I, and J. (A) The 3‐bottle choice test, (B) Total fluid intake over 72 h during a 3‐bottle choice test (*p* = 0.0303), (C) Solution preference as a percentage during a 3‐bottle choice test (*p* = 0.0203), (D) Elevated plus maze (EPM), (E,F): Duration spent in the open arms of an EPM, (G) Open field test, (H–J) Open field test (OFT) outcomes for mice maintained on the HFD, including total distance travelled (H), proportion of time spent in the center zone (I), and proportion of time spent in the outer zone (J), (K–M) OFT outcomes, including total distance travelled (K), proportion of time spent in the center zone (L), and proportion of time spent in the outer zone (M). Graphs show mean ± SEM. Panel C was analyzed using a two‐way repeated measures ANOVA while all other panels were analyzed using one‐way ANOVAs. ∗*p* < 0.05, ∗∗*p* < 0.01. Black: chow‐fed controls (HFD: *n* = 4, LFD: *n* = 20 for panels B, C, and F and *n* = 9 for panels K, L, and M), light blue: DIO‐sham‐operated group (HFD: *n* = 6, LFD: *n* = 17 for panels B, C, and F and *n* = 8 for panels K, L, and M), dark blue: DIO‐VSG‐operated group (HFD: *n* = 7, LFD: *n* = 23 for panels B, C, and F and *n* = 11 for panels K, L, and M). Detailed statistical analyses, including main effects, interaction effects, and post hoc comparisons, are provided in Table S4.

To assess the impact of VSG on reward‐related behavior, we used a 3‐bottle choice test (Figure 4A) where mice in the LFD‐maintained cohort got access to sucrose, saccharin, and water, with sucrose and saccharin being used to discriminate between the effect of VSG on the preference for nutritive versus non‐nutritive sweeteners. Total fluid intake was slightly higher in VSG‐ and sham‐operated mice compared to CF‐controls (Figure 4B, *P*_Treatment_ = 0.0303). Mice from all three treatment groups showed greater preference for sucrose and saccharin over water (Figure 4C, *P*_Treatment_ = 0.0203, *P*_Solution_ < 0.0001). We also assessed the impact of VSG surgery on anxiety‐like behavior using two established paradigms, the EPM (Figure 4D) and OFT (Figure 4G). These tests revealed no significant differences in time spent in the open arms of the EPM (Figure 4E,F). Analysis of the OFT showed no differences between groups in total locomotor activity or in the proportion of time spent in the center and outer zones under either HFD (Figure 4H–J) or LFD conditions (Figure 4K–M), indicating comparable exploratory behavior and anxiety‐like responses across all treatment groups. Together, these findings suggest that VSG does not substantially alter basal anxiety‐like behavior or general locomotor activity in male mice under either diet condition.

### 3.3 The Impact of VSG on Motivated Behavior and Cognitive Flexibility

**FIGURE 5:**
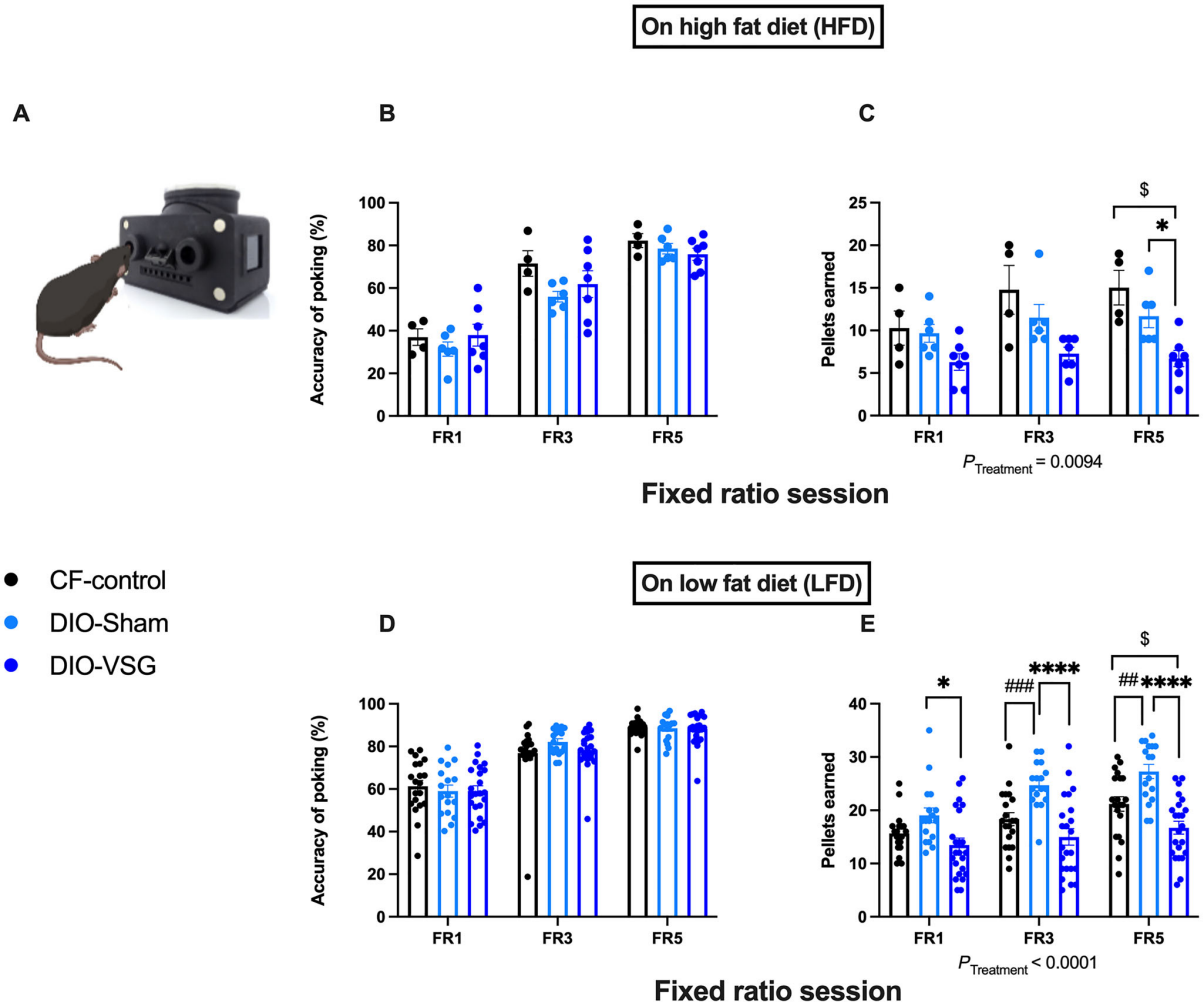
Effects of VSG on pellet retrieval and response accuracy during fixed ratio (FR) operant training. VSG‐, sham‐operated, and control mice were trained under fixed ratio schedules (FR1, FR3, and FR5; requiring 1, 3, or 5 responses per pellet, respectively) for three consecutive days per schedule prior to progressive ratio and reversal learning tasks. HFD—panels B and C and LFD—panels D and E. (A) Mouse performing nose poke, (B) The accuracy of poking, (C) Pellets earned (*p* = 0.0094), (D) The accuracy of poking, (E) Pellets earned (*p* < 0.0001). Graphs show mean ± SEM. All panels were analyzed using two‐way repeated measures ANOVAs. ∗*p* < 0.05, ∗∗*p* < 0.01, * = DIO‐VSG versus DIO‐sham‐operated, $ = DIO‐VSG versus chow‐fed control; # = DIO‐sham‐operated versus Chow‐fed control. Black: chow‐fed controls (HFD: *n* = 4, LFD: *n* = 20), light blue: DIO‐sham‐operated group (HFD: *n* = 6, LFD: *n* = 17), dark blue: DIO‐VSG‐operated group (HFD: *n* = 7, LFD: *n* = 23). Detailed statistical analyses, including main effects, interaction effects, and post hoc comparisons, are provided in Table S5.

To assess the impact of VSG on motivated behavior, mice first underwent fixed ratio (FR) operant training, which evaluates the ability to acquire rewards under increasing response requirements. All three treatment groups were tested under both HFD) and LFD conditions. Accuracy was comparable between groups in both the HFD‐ and LFD‐maintained experiments (Figure 5B,D). In the HFD study, DIO sham and VSG mice generally earned fewer pellets than controls at FR1–FR3 schedules (Figure 5C, *P*_Treatment_ = 0.0094). At FR5, VSG mice tended to earn fewer pellets than both sham‐operated and control mice, although these differences were modest (Figure 5C, *P*_DIO‐VSG vs. CF‐control_ = 0.0401, *P*_DIO‐sham vs. DIO‐VSG_ = 0.0333). In the LFD study, VSG mice generally earned fewer pellets than sham‐operated mice at all FR schedules (Figure 5E, *P*_Treatment_ < 0.0001, *P*_DIO‐sham vs. DIO‐VSG_ = 0.0155). At FR3 and FR5, sham‐operated mice earned more pellets than both control (FR3: *P*_DIO‐sham vs. CF‐control_ = 0.0009; FR5: *P*_DIO‐sham vs. CF‐control_ = 0.0074) and VSG‐operated mice (*P*_DIO‐sham vs. DIO‐VSG_ < 0.0001), and at FR5, VSG mice earned fewer pellets than sham‐operated mice (*P*_DIO‐sham vs. DIO‐VSG_ < 0.0001) and showed a modest reduction compared to control mice (*P*_DIO‐VSG vs. CF‐control_ = 0.0489). These findings may indicate that VSG can reduce reward acquisition under demanding operant conditions, particularly at higher FR schedules, and that this effect may be influenced by dietary context. However, as the different dietary conditions were evaluated in two independent cohorts, comparisons between dietary conditions should be interpreted with caution.

**FIGURE 6:**
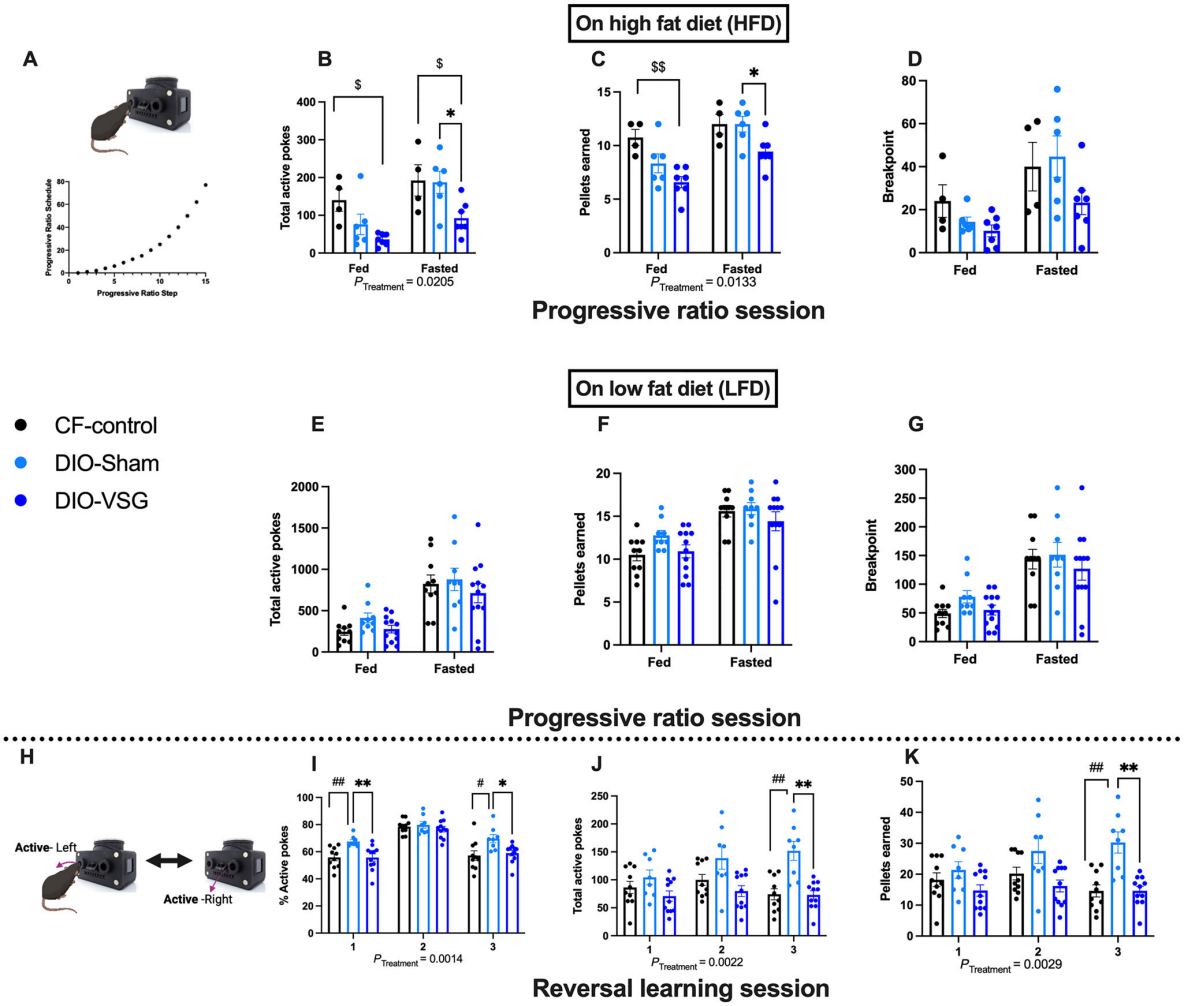
**T**he effect of VSG on motivated behavior and cognitive flexibility. VSG‐, sham‐operated, and control mice underwent a series of operant tests to investigate the effects of VSG on motivated behavior and cognitive flexibility whilst mice were maintained on HFD—panels B–D and LFD—panels E–K. (A) Progressive ratio task (PRT), (B) The total number of active pokes during the PRT (*p* = 0.0205), (C) The pellets earned during the PRT (*p* = 0.0133), (D) Breakpoint, (E–G) The total number of active pokes, total pellets earned, and breakpoint while on LFD respectively, (H) Reversal learning task (RLT), (I–K) The percentage of active pokes (*p* = 0.0014), the total number of active pokes (*p* = 0.0022), and the number of pellets earned (*p* = 0.0029) in the reversal learning task (RLT). Graphs show mean ± SEM. All panels were analyzed using two‐way ANOVAs. ∗*p* < 0.05, ∗∗*p* < 0.01, * = DIO‐VSG versus DIO‐sham‐operated, $ = DIO‐VSG versus chow‐fed control; # = DIO‐sham‐operated versus Chow‐fed control. Black: chow‐fed controls (HFD: *n* = 4, LFD: *n* = 10), light blue: DIO‐sham‐operated group (HFD: *n* = 6, LFD: *n* = 9 for panels E–G, *n* = 8 for panels I–K), dark blue: DIO‐VSG‐operated group (HFD: *n* = 7, LFD: *n* = 12 for panels E–G, *n* = 11 for panels I–K). Detailed statistical analyses, including main effects, interaction effects, and post hoc comparisons, are provided in Table S6.

To assess the impact of VSG on motivated behavior, we utilized a PRT which evaluates the willingness of the mouse to work for a palatable reward (Figure 6A). This task was completed using 20 mg sucrose pellets, in both mice maintained on HFD (Figure 6B–D) and those maintained on LFD (Figure 6E–G). The PRT was carried out both when mice had access to their food and following overnight food restriction. Mice that were maintained on HFD after undergoing VSG displayed a reduction in the number of pokes made to earn pellets (Figure 6B, *P*_Treatment_ = 0.0205, *P*_CF‐control (chow‐fed control) vs. DIO‐VSG (fed)_ = 0.0196, *P*_CF‐control vs. VSG (fasted)_ = 0.0255, and *P*_DIO‐sham vs. DIO‐VSG (fasted)_ = 0.0158), as well as a corresponding reduction in the number of pellets earned (Figure 6C, *P*_Treatment_ = 0.0133, *P*_CF‐control vs. DIO‐VSG (fed)_ = 0.0015, and *P*_DIO‐sham vs. DIO‐VSG (fasted)_ = 0.0297) in both fed and fasted states. While VSG‐operated mice also displayed lower breakpoints, this effect did not reach statistical significance (Figure 6D, *P*_Treatment_ = 0.0969).

Given that the breakpoint is the conventional measure of motivation in PRTs, these findings do not provide strong evidence for a reduction in motivation per se. However, VSG‐operated mice exhibited reduced operant responding, reflected by fewer nose pokes and the corresponding reduction in pellets earned. This suggests diminished reward‐directed responding under these testing conditions. Interestingly, mice maintained on LFD after undergoing VSG did not display these significant reductions in poke numbers (Figure 6E, *P*_Treatment_ = 0.3145) and corresponding number of pellets earned (Figure 6F, *P*_Treatment_ = 0.1573). Additionally, VSG did not significantly affect the breakpoint in mice maintained on the LFD (Figure 6G, *P*_Treatment_ = 0.3561). Together, these findings suggest that the reduction in operant responding observed after VSG may be influenced by dietary context. However, as the HFD and LFD conditions were evaluated in independent cohorts, the contribution of dietary context to these observations cannot be determined directly from this study. Furthermore, the HFD‐maintained cohort was relatively small and exploratory in nature, and therefore these findings should be interpreted cautiously and require further investigation in larger cohorts.

Cognitive flexibility was assessed via the RLT, which involved switching the active reward port across three sessions (Figure 6H). During the initial reversal, sham‐operated mice exhibited higher percentages of active pokes and earned more pellets than VSG and CF‐control mice (Figure 6I–K, *P*_Treatment_ = 0.0014 and *P*_Reversal step_ <0.0001, *P*_Treatment_ = 0.0022 and *P*_Treatment_ = 0.0029, respectively). This performance was not maintained during the second reversal, where the active port was consistent with the side the mice were initially trained on. However, in the third reversal, sham‐operated mice again exhibited higher percentages of active pokes and earned more pellets compared to VSG and CF‐control mice. (Figure 6H, *P*_Reversal step_ = 0.0242, *P*_Reversal step*Surgery_ = 0.0012; Figure 6I, *P*_Reversal step_ = 0.0040, *P*_Reversal step*Surgery_ = 0.0012). Sham‐operated mice that underwent laparotomy and post‐surgical recovery before transitioning to the LFD performed better in the RLT than both VSG‐operated and non‐surgical CF‐control mice. However, this observation was unexpected and, given the relatively small sample size of the reversal learning cohort, should be interpreted cautiously and requires replication before firm conclusions can be drawn.

## 4 Discussion

The present findings suggest that dietary context may influence motivation and cognitive flexibility following VSG, raising the possibility that metabolic interventions may affect higher‐order behavioral regulation. While VSG is well established to induce weight loss and other metabolic outcomes as reported in preclinical (Chambers et al. 2012; Stefater et al. 2011; Stefater et al. 2010) and clinical (Salminen et al. 2018) studies, our data extend beyond these canonical outcomes by demonstrating that reward‐related behaviors were reduced in VSG‐treated mice maintained on a HFD but not in a separate cohort transitioned to a LFD, and that cognitive flexibility does not uniformly improve following surgical weight loss. This focus on behavioral and cognitive adaptations aligns with emerging evidence that outcomes following bariatric interventions extend beyond metabolic improvements to include changes in psychological and behavioral domains such as body image, self‐esteem, and quality of life (AboKhozima et al. 2025). Together, these findings support a more integrative framework in which both diet and surgery may contribute to shaping not only energy balance, but also motivation, cognitive flexibility, and behavioral regulation.

The present findings reinforce and extend our understanding of the metabolic and behavioral consequences of VSG previously reported in preclinical (Chambers et al. 2012; Stefater et al. 2011; Stefater et al. 2010) and clinical (Salminen et al. 2018) studies. VSG reduced body weight, adiposity, and caloric intake, with some evidence of improved glucose handling in AUC analyses. Interestingly, CF‐control mice exhibited slightly reduced glucose tolerance compared to DIO‐sham and DIO‐VSG mice. Although counterintuitive, this may reflect differences in handling and habituation across cohorts. DIO mice underwent repeated daily handling, particularly during the post‐surgical recovery period, and were therefore extensively acclimatized to experimental procedures. In contrast, CF‐controls did not undergo surgery and experienced comparatively less procedural handling, which may have resulted in an increased stress response during metabolic testing. Acute stress is known to elevate circulating glucose levels via activation of the hypothalamic–pituitary–adrenal axis, potentially contributing to the higher glucose excursions observed in CF controls. However, HPA‐axis activity was not measured in the present study and this explanation remains speculative. Importantly, these mice were not weight matched and did not undergo surgical procedures, limiting direct metabolic comparability with the surgical cohorts. These findings should therefore be interpreted cautiously. These changes were further amplified by dietary modulation, with the transition to an LFD producing additional weight loss in both VSG and sham‐operated mice. Notably, in the LFD‐transitioned cohorts, all three treatment groups exhibited reductions in body weight. Sham‐operated mice showed the greatest weight loss, even exceeding that of VSG‐operated mice, despite having comparable caloric intake. This likely reflects several factors: differences in palatability between the HFD and LFD, which may have influenced energy intake and feeding behavior; and the possibility that VSG‐operated animals have a lower baseline energy requirement due to metabolic adaptations post‐surgery (Stefanidis et al. 2023), making them less prone to further weight loss. The switch to a LFD would have resulted in an energy deficit for both VSG‐ and sham‐operated mice, which had been consuming HFD for an extended period. Additionally, the lower cumulative LFD intake of VSG‐ and sham‐operated mice compared to CF‐controls likely contributed to further weight loss. This deficit was likely relatively greater in sham‐operated mice, as they lacked the caloric restriction imposed by VSG, resulting in more pronounced weight loss compared to VSG‐operated mice. This transient negative energy balance could have contributed in part to the variability in motivation and cognitive flexibility (Beckner et al. 2023) observed in FED3 tasks, particularly in sham‐operated mice. However, considering that the motivation and cognitive flexibility tasks were conducted at (day 36–47 after LFD‐transition), after body weights across all groups had begun to converge, cognitive outcomes were unlikely to be substantially influenced by acute differences in negative energy balance. This convergence is consistent with prior studies suggesting that dietary composition alone can drive substantial weight loss in mice, particularly as the mice advance in age (Corsetti et al. 2018). Consequently, some early metabolic and behavioral differences that were more apparent soon after surgery may diminish over time (Tindle et al. 2010; Guerrero‐Hreins et al. 2021; Cornejo‐Pareja et al. 2019; Gao et al. 2025), highlighting the importance of distinguishing between early versus late effects.

Importantly, these metabolic shifts were accompanied by increased interscapular temperature, used as a proxy of thermogenic activity in BAT, supporting emerging evidence that VSG enhances energy expenditure in mice (Stefanidis et al. 2023; Sandoval and Patti 2023). Given that BAT activation has been mechanistically linked to improvements in glucose metabolism and whole‐body insulin sensitivity (Rizzello et al. 2010; Brzozowska et al. 2023; Khalaf and Taegtmeyer 2013), our findings reinforce the evidence that VSG initiates compensatory endocrine and autonomic responses that promote increased energy expenditure in mice. The absence of a comparable decrease in inguinal WAT temperature following VSG, unlike in sham‐operated mice, together with increased interscapular temperature, suggests that VSG may modulate adipose tissue temperature regulation and BAT‐related thermogenic activity.

Behaviorally, our findings reflect the complexity and individual variability of reward‐related and affective outcomes following VSG, consistent with the heterogeneity observed in both rodent and human studies (Ahmed et al. 2018; Ribeiro et al. 2021; Sandoval 2019; Søndergaard Nielsen et al. 2018). While some clinical reports suggest metabolic surgery may shift hedonic preferences in humans, particularly toward non‐caloric sweeteners (Ahmed et al. 2018; Al‐Najim et al. 2018; Bueter et al. 2011), our data indicates that VSG does not significantly alter sucrose and saccharin preference. Our findings align with several rodent studies that fail to detect consistent changes in sweet taste preference after VSG, suggesting that the relationship between metabolic surgery and reward‐related ingestive behavior is complex and influenced by dietary background, post‐ingestive feedback, and perhaps strain‐specific factors (Nance et al. 2020; Qadhi et al. 2023).

Motivated behaviors, assessed using the PRT, revealed one of the more robust behavioral outcomes in this study. VSG‐treated mice maintained on a HFD exhibited reduced operant responding, reflected by fewer nose pokes and the associated reduction in pellets earned compared to sham‐operated mice, highlighting a decrease in sucrose‐seeking behavior. Although the breakpoints were also lower in VSG‐treated mice, this effect did not reach statistical significance and should be interpreted as a trend rather than a definitive effect. These findings are consistent with previous reports showing that metabolic surgery, including VSG and Roux‐en‐Y gastric bypass, can reduce mesolimbic dopamine activity and alter food reward processing through increased central GLP‐1 or PYY signaling and downstream modulation of mesolimbic pathways (Hankir et al. 2018; Hamamah et al. 2024; Reddy et al. 2014).

Importantly, this reduction in operant responding behavior was not observed when VSG‐operated animals were transitioned to a LFD, with their performance resembling that of sham‐operated mice. This raises the possibility that post‐surgical motivational deficits may vary based on dietary context. While these findings highlight the possibility that dietary context influences post‐surgical motivation and cognitive flexibility, this study was not designed to directly assess this relationship. Therefore, it warrants further investigation through studies specifically designed to assess how dietary context affects the behavioral outcomes following VSG. Dietary fat content is known to modulate dopaminergic tone and reward‐related behavior (Wallace and Fordahl 2022; Darcey et al. 2023; Décarie‐Spain et al. 2016), and VSG may interact with this effect to influence behavioral outputs. In clinical studies, patients who maintain a structured, nutrient‐dense diet post‐operatively report reduced cravings, at least during the initial post‐operative stage (Koball et al. 2024; Leahey et al. 2011). Conversely, re‐exposure to energy‐dense, palatable foods appears to be associated with blunted reward responses (Martinou et al. 2022; Nansel et al. 2016). This effect may paradoxically drive compensatory eating behaviors, specially where typically rewarding fatty and sugary foods are concerned, in an attempt to recapture the pleasurable sensations that were previously associated with these types of food (Tolvanen et al. 2023). The increased consumption of sugary and fatty food may arise in the form of grazing and/or binge‐like eating patterns, potentially underpinning in long‐term weight regain (Leahey et al. 2011; Tolvanen et al. 2023). These maladaptive behaviors are thought to be driven by the interplay between neuroendocrine adaptations, such as altered gut peptide levels and dopamine signaling, environmental factors, such as the accessibility of energy‐dense, palatable food, and also, unhealthy dietary habits from before undergoing metabolic surgery (Halbout et al. 2019; Liljeholm and O'Doherty 2012; Leahey et al. 2011).

A dissociation between motivational and cognitive outcomes was also evident. In the RLT, sham‐operated mice demonstrated enhanced cognitive flexibility relative to both VSG and CF‐control groups. These observations suggest that weight loss associated with the transition to the LFD may contribute to improved executive function. However, as sham‐operated mice also underwent surgery and recovery, the effects of dietary intervention cannot be separated from surgery‐related influences. In contrast, VSG‐treated mice did not display such enhancements, despite experiencing equivalent or greater weight loss. This divergence implies that while surgical and non‐surgical routes to weight loss may yield similar metabolic outcomes, they may engage distinct neuronal pathways. While clinical studies have reported improvements in memory, attention, and executive function following metabolic surgery (Nota et al. 2020; Gunstad et al. 2011), such effects are variable across patient groups (Vreeken et al. 2023). Our data indicate that surgical weight loss does not inherently promote improved cognitive flexibility, at least in the context of the reversal learning paradigm used here. The observation that sham‐operated mice performed better than VSG‐operated and CF‐control mice was not anticipated. Given the relatively small sample size of the cohort that underwent the RLT, this should be interpreted cautiously and requires replication before firm conclusions can be made. Preclinical studies exploring similar tasks have also yielded mixed results, with some showing cognitive improvements post‐surgery (Alosco et al. 2014) and others reporting no change or even adverse effects depending on age, sex, or type of procedure (Roy et al. 2024; Jumbe et al. 2017).

Measures of anxiety‐like behavior and locomotor activity (EPM, OFT) did not differ significantly between groups. While this might reflect a lack of impact of VSG on these behaviors, it may also reflect the limitations of acute behavioral assays in modeling complex psychological outcomes (Gencturk and Unal 2024). Clinical studies have reported both improvements and new‐onset psychiatric symptoms post‐VSG, often with a delayed trajectory (Castaneda et al. 2019; Backman et al. 2016). The observed pattern in our mice may mirror this complexity. Notably, human studies suggest that psychiatric outcomes post‐surgery are influenced by psychosocial context, dietary patterns, and prior history (Jumbe et al. 2017), factors that cannot be easily modeled in short‐term rodent experiments. Furthermore, longitudinal behavioral studies in mice are further complicated by task familiarity and repeated testing, sometimes requiring the use of independent cohorts to mitigate the influence of repeated exposure.

Several limitations should be acknowledged. Behavioral testing occurred during a period of ongoing weight change and transient negative energy balance following dietary transition, both of which may have contributed to the motivational and cognitive differences observed between treatment groups. CF‐controls did not receive sham surgery, limiting direct comparability for some metabolic analyses. The lack of pre‐ and post‐surgical within‐subject measurements reduced the ability to perform paired statistical analyses. Additionally, only male mice were studied to minimize variability introduced by the estrous cycle and maintain consistency with prior work in this model; therefore, future studies should include female cohorts to evaluate sex‐specific responses. Sample size and task‐specific sensitivity may constrain the detection of subtle behavioral differences, and FED3 outcomes may be influenced by prior learning history, stress, and energy status. Specifically, experiments conducted in mice maintained on HFD had small sample sizes, as these were intended as pilot studies before progressing to a study design that better mimics the real‐world clinical situation, where patients are advised to adopt a low‐calorie diet post‐surgery. A further limitation is that our interpretation of diet‐dependent effects on post‐surgical motivation is based on comparisons between separately run dietary cohorts and does not represent a formal Diet × Surgery interaction within a single integrated model. As such, while the observed pattern is consistent with the possibility that dietary context influences motivational and reward‐related outcomes after VSG, this finding should be interpreted cautiously. Additionally, multiple behavioral domains were assessed across the study, increasing the risk of type I error when findings are considered collectively. While each behavioral test was utilized to assess a distinct, pre‐defined construct and was independently analyzed, no formal correction for multiple comparisons was applied across the full behavioral battery. Thus, although some behavioral outcomes reached statistical significance, the increased risk of type I error means that these findings should be interpreted with caution and warrant replication in independent cohorts.

 Future studies should incorporate longitudinal designs, explicitly test for the interaction between diet and surgery and include female cohorts to improve translational relevance. Additionally, in the future, the neurobiological mechanisms underlying these diet‐ or surgery‐dependent mechanisms should be investigated. In particular, alterations in mesolimbic dopamine signaling in key reward‐related brain regions such as the nucleus accumbens (NAc). For example, monitoring of real‐time dopamine release using fiber photometry (Simpson et al. 2024) or chemogenetic approaches to manipulate specific neural circuits can help determine whether mesolimbic dopamine signaling within reward‐related regions such as the NAc contributes to these behavioral effects. Additionally, GLP‐1 receptor signaling within prefrontal cortical regions has been suggested to modulate cognitive control, reward processing, and motivation (Gao et al. 2026; Eren‐Yazicioglu et al. 2020) and therefore, potentially presents a pathway linking metabolic intervention and changes in reward‐related and behavioral processes. Elucidating these underlying mechanisms will be crucial to understanding how bariatric surgical interventions shape these outcomes.

In summary, this study confirms the robust metabolic benefits of VSG and provides new insight into its behavioral effects. VSG suppressed motivated sucrose seeking in mice maintained on an HFD, whereas this effect was not observed in a separate cohort transitioned to an LFD. Although these differing outcomes across dietary conditions suggest a potential role for dietary context in influencing behavioral outcomes following VSG, this requires confirmation via future studies designed to evaluate diet–surgery interactions. VSG also did not demonstrate the improvement in cognitive flexibility evident in sham‐operated mice. Further investigation is needed to clarify the mechanisms underlying the impact of sleeve gastrectomy on metabolic and behavioral outcomes. A deeper mechanistic understanding may help optimize surgical approaches, identify individuals at risk of suboptimal behavioral responses, and guide the development of adjunctive strategies to maximize therapeutic efficacy.

## Author Contributions

**Jayasinghe R**.: investigation, methodology, validation, visualization, formal analysis, project administration, data curation, writing – original draft preparation, writing – review and editing. **Piotrowski J**.: investigation, methodology, visualization, formal analysis, data curation. **Greaves E**.: investigation, project administration. **Lemus M. B**.: investigation, project administration. **Reed F**.: methodology, formal analysis, data curation. **Conn K**.: investigation, methodology, data curation. **Brown R. M**.: conceptualization, writing – review and editing. **Sumithran P**.: conceptualization, writing – review and editing. **Andrews Z. B**.: conceptualization, methodology, visualization, formal analysis, supervision, writing – review and editing. **Foldi C. J**.: conceptualization, investigation, methodology, validation, visualization, software, formal analysis, project administration, data curation, resources, supervision, writing – original draft preparation, writing – review and editing. **Oldfield B. J**.: conceptualization, investigation, funding acquisition, resources, supervision, writing – review and editing. **Stefanidis A**.: conceptualization, investigation, methodology, validation, visualization, software, formal analysis, project administration, data curation, funding acquisition, resources, supervision, writing – original draft preparation, writing – review and editing.

## Funding

This work was supported by the National Health and Medical Research Council (NHMRC) of Australia through Grant GNT1145142, awarded to Prof. Brian Oldfield at Monash University.

## Conflicts of Interest

The authors declare no conflicts of interest.

## Supporting information




**Supplementary Tables**: brb371766‐sup‐0001‐SuppMat.docx

## Data Availability

The data that support the findings of this study are available from the corresponding author upon reasonable request.
