# Supplementary material for: The Impact of Vertical Sleeve Gastrectomy on Behavioral and Reward‐Related Outcomes
Source: Brain Behav. 2026 Sep 14;16(9):e71766. doi: 10.1002/brb3.71766 (PMC13575494; doi:10.1002/brb3.71766)
Supplement: Supplementary file 1 [file BRB3-16-e71766-s001.docx]

**Supplementary Materials**

**Supplementary Table 1.** Statistical analyses for Figure 1.

| **Figure** | **Statistical Test** | **Group (n)** | **Main analysis result** | **Post-hoc multiple comparisons of interest** |
| --- | --- | --- | --- | --- |
| **1B** | Two-way Mixed effects ANOVA | DIO-sham n=6; DIO-VSG n=7 | Time F (66, 577) = 1.826, **p= 0.0002**  Treatment F (1,11) = 26.59, **p= 0.0003**  Interaction F (66, 577) = 7.205, **p< 0.0001** |  |
| **1C** |  |  | Time F (1.964, 20.40) = 1.822, p= 0.1874  Treatment F (1,11) = 66.49, **p< 0.0001**  Interaction F (49, 509) = 8.168, **p < 0.0001** |  |
| **1D** |  |  | Time F (2.106,23.17) = 1331, **p< 0.0001**  Treatment F (1, 11) = 21.60, **p= 0.0007**  Interaction F (66, 726) = 7.869, **p< 0.0001** |  |
| **1E** | Two-way RM ANOVA | CF-control n=5; DIO-sham n=6; DIO-VSG n=7 | Time F (2.218, 33.27) = 39.33, **p< 0.0001**  Treatment F (2, 15) = 2.596, **p= 0.1076**  Interaction F (8, 60) = 2.746, **p= 0.0119** | 60 minutes- DIO-VSG vs. CF-control: **p= 0.0430**  120 minutes- DIO-VSG vs. CF-control: **p= 0.0098**  DIO-sham vs. CF-control: **p= 0.0341** |
| **1F** | One-way ANOVA | CF-control n=5; DIO-sham n=6; DIO-VSG n=7 | F (2, 15) = 4.246, **p= 0.0346** | DIO-VSG vs. CF-control: **p= 0.0351** |
| **1G** | Two-way RM ANOVA | CF-control n=5; DIO-sham n=6; DIO-VSG n=7 | Time F (2.536, 38.03) = 16.86, **p< 0.0001**  Treatment F (2, 15) = 3.534, **p= 0.0552**  Interaction F (8, 60) = 3.345, **p= 0.0031** |  |
| **1H** | One-way ANOVA | CF-control n=5; DIO-sham n=6; DIO-VSG n=7 | F (2, 15) = 4.802, **p= 0.0244** | DIO-VSG vs. CF-control: **p= 0.0260** |

**Supplementary Table 2.** Statistical analyses for Figure 2.

| **Figure** | **Statistical Test** | **Group (n)** | **Main analysis result** | **Post-hoc multiple comparisons of interest** |
| --- | --- | --- | --- | --- |
| **2A** | One-way ANOVA | CF-control n=5; DIO-sham n=6; DIO-VSG n=7 | F (2, 15) = 18.31**, p< 0.0001** | DIO-sham vs. DIO-VSG: **p= 0.0002**  DIO-sham vs. CF-control: **p= 0.0004** |
| **2B** |  |  | F (2, 15) = 17.19, **p= 0.0001** | DIO-sham vs. DIO-VSG: **p= 0.0013**  DIO-VSG vs. CF-control: **p= 0.0002** |
| **2C** |  |  | F (2, 15) = 9.408, **p= 0.0023** | DIO-sham vs. DIO-VSG: **p= 0.0024**  DIO-VSG vs. CF-control: **p= 0.0221** |
| **2D** |  |  | F (2, 15) = 11.89, **p= 0.0008** | DIO-sham vs. DIO-VSG: **p= 0.0419**  DIO-sham vs. CF-control: **p= 0.0419**  VSG vs. CF-control: **p= 0.0006** |
| **2E** | **Two-way Mixed effects ANOVA** | CF-control n=4; DIO-sham n=6; DIO-VSG n=7 | Time F (1.555, 19.44) = 5.802, **p= 0.0153**  Treatment F (2, 13) = 12.09, **p= 0.0011**  Interaction F (4, 25) = 1.232, **p= 0.3226** | Week 1 DIO-sham vs. DIO-VSG: **p= 0.0034**  Week 2 DIO-sham vs. DIO-VSG: **p= 0.0334**  Week 3 DIO-sham vs. CF-control: **p= 0.0320**  Week 3 DIO-sham vs. DIO-VSG: **p= 0.0063** |
| **2F** |  |  | Time F (1.784, 22.29) = 1.098, p= 0.3445  Treatment F (2, 13) = 0.8771, p= 0.4392  Interaction F (4, 25) = 2.032, p= 0.1206 |  |
| **2G** |  |  | Time F (1.663, 21.62) = 0.06040, p= 0.9143  Treatment F (2, 13) = 7.012**, p= 0.0086**  Interaction F (4, 26) = 0.8289, p= 0.5189 | Week 1 DIO-sham vs. DIO-VSG: **p= 0.0032**  Week 1 DIO-sham vs. CF-control: **p= 0.0242** |
| **2H** |  |  | Time F (1.480, 19.24) = 0.2916, p= 0.6845  Treatment F (2, 13) = 1.443, p= 0.2716  Interaction F (4, 26) = 2.328, p= 0.0828 | Week 1 DIO-sham vs. CF-control: **p= 0.0459** |

**Supplementary Table 3.** Statistical analyses for Figure 3.

| **Figure** | **Statistical Test** | **Group (n)** | **Main analysis result** |
| --- | --- | --- | --- |
| **3B** | Two-way Mixed effects ANOVA | CF-control n=20; DIO-sham n=17; DIO-VSG n=23 | Time F (20, 673) = 58.65, **p< 0.0001**  Treatment F (1, 38) = 15.51, **p= 0.0003**  Interaction F (20, 673) = 17.28, **p< 0.0001** |
| **3C** |  |  | Time F (1.011, 34.88) = 64.13, **p< 0.0001**  Treatment F (1, 38) = 31.04, **p< 0.0001**  Interaction F (18, 621) = 17.51, **p< 0.0001** |
| **3D** |  |  | Time F (28, 1064) = 993.9, **p< 0.0001**  Treatment F (1, 38) = 28.99, **p< 0.0001**  Interaction F (28, 1064) = 21.74, **p< 0.0001** |
| **3E** |  |  | Time F (27, 1539) = 21.37, **p< 0.0001**  Treatment F (2, 57) = 13.04, **p< 0.0001**  Interaction F (54, 1539) = 10.38, **p< 0.0001** |
| **3F** |  |  | Time F (27, 1539) = 19.00, **p< 0.0001**  Treatment F (2, 57) = 26.20, **p< 0.0001**  Interaction F (54, 1539) = 10.55, **p< 0.0001** |
| **3G** |  |  | Time F (67, 3752) = 10448, **p< 0.0001**  Treatment F (2, 56) = 42.70, **p< 0.0001**  Interaction F (134, 3752) = 13.19, **p< 0.0001** |

**Supplementary Table 4.** Statistical analyses for Figure 4.

| **Figure** | **Statistical Test** | **Group (n)** | **Main analysis result** |
| --- | --- | --- | --- |
| **4B** | One-way ANOVA | CF-control n=20; DIO-sham n=17; DIO-VSG n=23 | F (2, 57) = 3.719, **p= 0.0303** |
| **4C** | Two-way RM ANOVA | CF-control n=20; DIO-sham n=17; DIO-VSG n=23 | Solution F (1.163, 66.27) = 33.94, **p< 0.0001**  Treatment F (2, 57) = 4.176, **p= 0.0203**  Interaction F (4, 114) = 0.6084, p= 0.6574 |
| **4E** | One-way ANOVA | CF-control n=5; DIO-sham n=6; DIO-VSG n=7 | F (2, 14) = 0.1057, p=0.9004 |
| **4F** | One-way ANOVA | CF-control n=20; DIO-sham n=17; DIO-VSG n=23 | F (2, 57) = 1.172, p=0.3171 |
| **4H** | One-way ANOVA | CF-control n=5; DIO-sham n=6; DIO-VSG n=7 | F (2, 14) = 2.664, p=0.1046 |
| **4I** |  |  | F (2, 14) = 0.7830, p=0.4761 |
| **4J** |  |  | F (2, 14) = 0.3468, p=0.7128 |
| **4K** | One-way ANOVA | CF-control n=10; DIO-sham n=8; DIO-VSG n=11 | F (2, 26) = 0.1584, p=0.8543 |
| **4L** |  |  | F (2, 26) = 0.5292, p=0.5953 |
| **4M** |  |  | F (2, 26) = 0.4655, p=0.6330 |

**Supplementary Table 5.** Statistical analyses for Figure 5.

| **Figure** | **Statistical Test** | **Group (n)** | **Main analysis result** | **Post-hoc multiple comparisons of interest** |
| --- | --- | --- | --- | --- |
| **5B** | Two-way RM ANOVA | CF-control n=4; DIO-sham n=6; DIO-VSG n=7 | FR step F (1.520, 21.27) = 86.07, **p< 0.0001**  Treatment F (2, 14) = 1.733, p= 0.2125  Interaction F (4, 28) =0.9423, p= 0.4541 |  |
| **5C** |  |  | FR step F (1.767, 24.73) = 12.82, **p= 0.0002**  Treatment F (2, 14) = 6.635, **p= 0.0094**  Interaction F (4, 28) =2.790, **p= 0.0456** | FR5 DIO-sham vs. DIO-VSG: **p= 0.0333**  FR5 DIO-VSG vs. CF-control: **p= 0.0401** |
| **5D** | Two-way RM ANOVA | CF-control n=20; DIO-sham n=17; DIO-VSG n=23 | FR step F (1.909, 108.8) = 166.0, **p< 0.0001**  Treatment F (2, 57) = 0.1983, p= 0.8207  Interaction F (4, 114) =0.9292, p= 0.4497 |  |
| **5E** |  |  | FR step F (1.865, 106.3) = 43.84, **p< 0.0001**  Treatment F (2, 57) = 14.29, **p< 0.0001**  Interaction F (4, 114) =3.249, **p= 0.0145** | FR1 DIO-sham vs. DIO-VSG: **p= 0.0155**  FR3 DIO-sham vs. DIO-VSG: **p< 0.0001**  FR3 DIO-sham vs. CF-control: **p= 0.0009**  FR5 DIO-sham vs. DIO-VSG: **p< 0.0001**  FR5 DIO-VSG vs. CF-control: **p= 0.0489**  FR5 DIO- sham vs. CF-control: **p= 0.0074** |

**Supplementary Table 6.** Statistical analyses for Figure 6.

| **Figure** | **Statistical Test** | **Group (n)** | **Main analysis result** | **Post-hoc multiple comparisons of interest** |
| --- | --- | --- | --- | --- |
| **6B** | Two-way RM ANOVA | CF-control n=4; DIO-sham n=6; DIO-VSG n=7 | Feeding state F (1, 14) = 53.20, **p< 0.0001**  Treatment F (2, 14) = 5.199, **p= 0.0205**  Interaction F (2, 14) = 3.847, **p= 0.0466** | Fed DIO-VSG vs. CF-control: **p= 0.0196**  Fasted DIO-sham vs. DIO-VSG: **p= 0.0158**  Fasted DIO-VSG vs. CF-control: **p= 0.0255** |
| **6C** |  |  | Feeding state F (1, 14) = 138.2, **p< 0.0001**  Treatment F (2, 14) = 5.969, **p= 0.0133**  Interaction F (2, 14) = 9.033, **p= 0.0030** | Fed DIO-VSG vs. CF-control: **p= 0.0015**  Fasted DIO-sham vs. DIO-VSG: **p= 0.0297** |
| **6D** |  |  | Feeding state F (1, 14) = 16.12, **p= 0.0013**  Treatment F (2, 14) = 2.770, p= 0.0969  Interaction F (2, 14) = 1.317, p= 0.2991 |  |
| **6E** | Two-way RM ANOVA | CF-control n=10; DIO-sham n=9; DIO-VSG n=12 | Feeding state F (1, 28) = 50.32, **p< 0.0001**  Treatment F (2, 28) = 1.206, p= 0.3145  Interaction F (2, 28) = 0.4023, p= 0.6726 |  |
| **6F** |  |  | Feeding state F (1, 28) = 38.73, **p< 0.0001**  Treatment F (2, 28) = 1.977, p= 0.1573  Interaction F (2, 28) = 0.9112, p= 0.4136 |  |
| **6G** |  |  | Feeding state F (1, 28) = 48.83, **p< 0.0001**  Treatment F (2, 28) = 1.072, p= 0.3561  Interaction F (2, 28) = 0.4140, p= 0.6650 |  |
| **6I** | Two-way RM ANOVA | CF-control n=10; DIO-sham n=8; DIO-VSG n=11 | Reversal number F (1.860, 48.36) = 57.31, **p< 0.0001**  Treatment F (2, 26) = 8.592, **p= 0.0014**  Interaction F (4, 52) = 1.846, p= 0.1340 | Reversal 1 DIO-sham vs. DIO-VSG: **p= 0.0046**  Reversal 1 DIO-sham vs. CF-control: **p= 0.0023**  Reversal 3 DIO-sham vs. DIO-VSG: **p= 0.0289**  Reversal 3 DIO-sham vs. CF-control: **p= 0.0394** |
| **6J** |  |  | Reversal number F (1.961, 51.00) = 6.239, **p= 0.0040**  Treatment F (2, 26) = 7.816, **p= 0.0022**  Interaction F (4, 52) = 5.282, **p= 0.0012** | Reversal 3 DIO-sham vs. DIO-VSG: **p= 0.0047**  Reversal 3 DIO-sham vs. CF-control: **p= 0.0055** |
| **6K** |  |  | Reversal number F (1.993, 51.81) = 4.009, **p= 0.0242**  Treatment F (2, 26) = 7.374, **p= 0.0029**  Interaction F (4, 52) = 5.255, **p= 0.0012** | Reversal 3 DIO-sham vs. DIO-VSG: **p= 0.0055**  Reversal 3 DIO-sham vs. CF-control: **p= 0.0057** |
